# Phase distribution regulation of formamidinium-based quasi-2D perovskites through solution engineering†

**DOI:** 10.1039/d4tc02231a

**Published:** 2024-08-22

**Authors:** Xiao Zhang, Lisanne Einhaus, Annemarie Huijser, Johan E. ten Elshof

**Affiliations:** a Inorganic Materials Science Group, MESA+ Research Institute, University of Twente 7500 AE Enschede The Netherlands j.e.tenelshof@utwente.nl x.zhang-6@utwente.nl; b PhotoCatalytic Synthesis Group, MESA+ Research Institute, University of Twente 7500 AE Enschede The Netherlands l.m.einhaus@utwente.nl j.m.huijser@utwente.nl

## Abstract

Quasi-2D perovskites have attracted attention as potential solar energy absorber materials due to their balanced efficiency and stability and their unique quantum-well structures. In order to facilitate directional excitons and charge carrier transport and preferential energy transfer landscape in photovoltaic thin films, the phase distribution formed by different types of microstructural domains should be regulated. In this work, the Dion–Jacobson-type spacer 1,4-phenylenedimethanammonium (PDMA) was used, and different strategies were pursued to control the phase distribution in formamidinium-based (FA) quasi-2D perovskites based on the composition of (PDMA)FA_4_Pb_5_I_16_. In general, doping with FACl modulated the crystallization kinetics, forming 2D low-*n* crystals on the top surface or a reversed-gradient phase distribution, depending on whether excess or substitutional doping was employed. Alternatively, mixing with a Ruddlesden–Popper spacer helped bridging to adjacent octahedra in pure PDMA-based perovskites and improved crystallization, while regulating the quantum-well structures to give a normal-gradient phase distribution, where 2D domains resided on the bottom side. By combining FACl doping and spacer mixing, the film showed both a reversed-gradient phase distribution and larger vertically aligned grains. This work contributes to the knowledge of how to manipulate and regulate the phase distribution in FA-based quasi-2D perovskites and further paves the way for fabricating corresponding devices with high efficiency and stability.

## Abstract 2

Mixing a Dion–Jacobson spacer and a Ruddlesden–Popper spacer regulates quasi-2D perovskite thin films for a normal-gradient phase distribution, while FACl additive doping regulates the films to give a reversed-gradient phase distribution.

## Graphical abstract

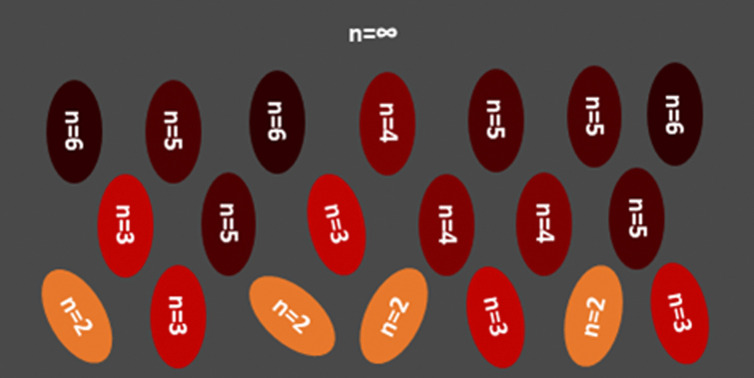

## 1. Introduction

Metal halide perovskites (MHPs) are currently one of the most popular solar energy absorber materials used to boost photovoltaic performance. Among the different types of MHPs, two-dimensional (2D) perovskites have attracted extensive attention in more recent years, since they comprise hydrophobic organic spacers which provide higher long-term stability against external stimuli under operational conditions, such as moisture, heat, oxygen and ion migration, compared to their 3D analogues.^1–11^ Although the power conversion efficiency (PCE) of 2D perovskites is still lagging behind that of the 3D perovskites, the record PCE of formamidinium (FA)-based 2D perovskite solar cells (PSCs) has reached over 20%, achieving a balanced efficiency and intrinsic stability.^12^

**Fig. 1:**
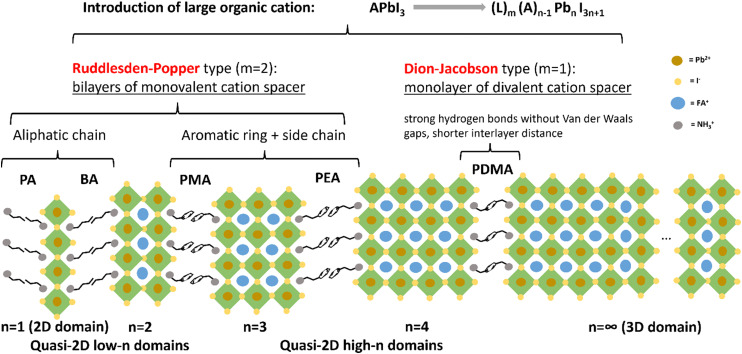
Schematic illustration of the structure and phase distribution of quasi-2D perovskites with Ruddlesden–Popper-type and Dion–Jacobson-type spacers.

As depicted in Fig. 1, by introducing large organic spacers along the (100) plane of 3D perovskites,^13–15^ 2D and quasi-2D perovskites with a chemical formula of (L)_*m*_(A)_*n*−1_(B)_*n*_(X)_3*n*+1_ are formed, where L represents the large organic spacers, A are small monovalent cations (*e.g.*, methyl ammonium MA^+^, FA^+^, and Cs^+^) fitting in the [BX_6_]^4−^ octahedral frameworks, B are divalent cations (*e.g.*, Pb^2+^ and Sn^2+^), X are halide ions (*e.g.*, I^−^, Br^−^, and Cl^−^), *m* depends on the spacer type (*m* = 2 for a monovalent Ruddlesden–Popper spacer and *m* = 1 for a divalent Dion–Jacobson spacer), and *n* represents the average number of inorganic layers between two organic spacers (*n* = 1 for pure 2D perovskites, *n* ≥ 2 for quasi-2D perovskites, and *n* = ∞ for 3D perovskites).^16–21^ Since the value of *n* can vary locally on a microscale, we employ the term 〈*n*〉 further on to denote the global average value in this paper.

In our work, the Dion–Jacobson (DJ) spacer 1,4-phenylenedimethanammonium (PDMA) was primarily used as the L-site spacer. Other Ruddlesden–Popper (RP) spacers, such as propylammonium (PA), *n*-butylammonium (BA), allylammonium (ALA), phenylmethylammonium (PMA), and phenethylammonium (PEA), were also used for comparison or for mixing with PDMA. FA was used as the A-site cation, since FA-based perovskites have higher thermal stability than the methylammonium (MA) series, owing to the higher sublimation temperature of FA compared with that of MA.^22^ Moreover, FA-based perovskites have a smaller bandgap than the MA series because of the increasing Pb–I–Pb angles induced by the larger size of FA, thus covering a larger part of the solar spectrum.^23–26^ Therefore, FA-based perovskites have higher potential to generate solar cells with both high efficiency and high thermal stability. However, due to the large size of FA, the Goldschmidt tolerance factor of the FAPbI_3_ perovskite structure approaches its maximum value 1, beyond which the cubic phase with corner-shared octahedra is not structurally stable.^27,28^ In fact, FAPbI_3_ has multiple polymorphic phases, the photo-inactive hexagonal δ-phase, or the yellow phase, of which is the stable phase at room temperature. The phase transition into the desired cubic α-phase, or the black phase, takes place at higher temperature (150–185 °C).^29,30^ The use of *N*-methyl-2-pyrrolidone (NMP) as a co-solvent together with the primary solvent dimethyl formamide (DMF) can yield a stable α-phase FAPbI_3_. NMP has a weak intermolecular interaction with DMF but strongly coordinates with PbI_2_ and FAI. The as-formed PbI_2_-NMP intermediate phase can directly convert into the α-phase after annealing.^31^

Additive doping is another effective way to form α-phase FAPbI_3_. Chloride salt derivatives (*e.g.* MACl, FACl, and PbCl_2_) can suppress the formation of the δ-phase and support the formation of the α-phase, regulating the crystal growth orientation.^32–34^ By incorporating *N,N*-dimethylimidodicarbonimidic diamide hydroiodide (DIAI), the intermediate phase FAI–PbI_2_–dimethyl sulfoxide (DMSO) complex is eliminated, so that the δ-phase is directly converted into the desired phase α-FAPbI_3_.^35^ Bu *et al.* used both the co-solvent systems DMF/NMP and MACl as additives to grow high-quality α-phase FA-based 3D perovskite solar cells with a PCE of 24.02%.^36^ However, a comprehensive understanding of what controls the crystallization, and especially the phase distribution in quasi-2D perovskites, is still lacking.

For quasi-2D perovskite thin films fabricated with the solution method with a certain 〈*n*〉 value, there are typically multiple local microstructural domains of different *n* values coexisting, where low-*n* domains (*n* = 1, 2) and high-*n* domains (*n* = 4, 5…) are heterogeneously distributed or concentrated in different areas within the film. The multiple quantum-well structures form a so-called phase distribution, which determines the charge carrier transport pathways and energy transfer landscapes.^37–43^ Different phase distributions (*e.g.* normal-gradient with low-*n* domains at the bottom and high-*n* domains at the top of the film, or a reversed-gradient with the reversed distribution of low-*n* and high-*n* domains) are suitable for different solar cell configurations (*e.g.* p–i–n where the hole transport layer is at the bottom interface and the electron transport layer is at the top interface, or the n–i–p configuration) due to different energy level alignments. Therefore, knowing how to tune the phase distribution of quasi-2D perovskites is of vital importance.

Our work aims at fabricating stable α-phase FA-based quasi-2D perovskites with controlled phase distribution. Through FACl doping, by both excess and substitutional doping, the phase distribution can be regulated into a reversed-gradient manner, enabling steering of the directionality of excitons and charge transport, and protecting the film from moisture penetration. Spacer engineering was also carried out by mixing the Dion–Jacobson spacer PDMA and two Ruddlesden–Popper spacers, ALA and PA. The phase distribution of *n*-domains can be further regulated into either a similar reversed-gradient or a normal-gradient manner, facilitating an energy transfer cascade, favouring directional excitons and charge transport and a potential electron tunnelling through the interlayer,^44^ thus paving the way towards future devices with high efficiency and stability.

## 2. Results and discussion

### 2.1. Fabrication of stable α-phase quasi-2D perovskites (PDMA)(FA)_4_Pb_5_I_16_

**Fig. 2:**
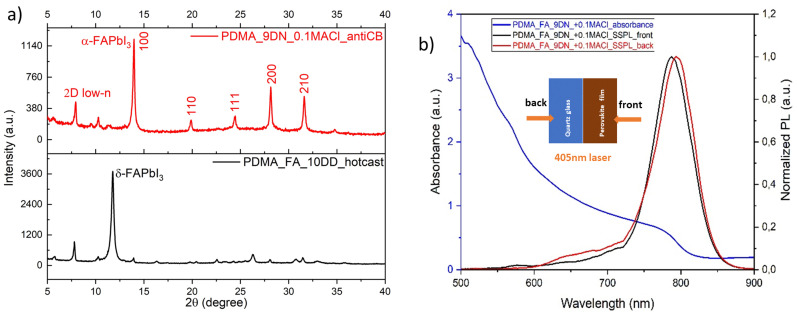
The crystallography and phase distribution of quasi-2D perovskites (PDMA)(FA)_4_Pb_5_I_16_. (a) Powder X-ray diffractograms of δ-phase and α-phase perovskites made from different precursor solution inks and processing methods. (b) UV-vis absorption spectra and steady-state photoluminescence from both front (perovskite) and back (substrate) sides of the quasi-2D perovskite thin films with 〈*n*〉 = 5. The inset shows the illumination directions.

Following the same fabrication conditions as with the MA series in our previous work^45^, *i.e.* using a 10 : 1 volume ratio of DMF : DMSO as the solvent mixture, using the hot-casting processing method and post-annealing at 100 °C, the quasi-2D perovskite (PDMA)(FA)_4_Pb_5_I_16_ showed the photo-inactive yellow phase or δ-phase (Fig. 2a), which is the stable phase at room temperature for FAPbI_3_. The phase transition into a corner-shared cubic phase can only occur by providing more thermal energy. Therefore, we increased the post-annealing temperature to 150 °C. Since the hot-casting method by pre-heating the substrate only changed the reaction kinetics, but not intermediate chemistry, we changed the process to the anti-solvent method, by dripping chlorobenzene (CB) onto the as-spin-coated perovskite precursor solution, to induce a fast supersaturation state and solvent extraction. Then, we conducted an orthogonal experiment to investigate which solvent combination and additive ratios could produce a stable black phase or α-phase. For the solvent mixture, we selected 4 : 1 (v/v) DMF : DMSO and 9 : 1 (v/v) DMF : NMP, while for additive doping, we selected MACl and ammonium thiocyanate (NH_4_SCN) in different molar ratios with respect to the constant Pb^2+^ concentration or a mixture of the two (Table S1, ESI†). The results are shown in Fig. 2a and Fig. S1 (ESI†), with 9 : 1 (v/v) DMF : NMP as the solvent combination and 10 mol% of excess MACl addition indicated a clean α-phase without other phase impurities, prevailing other solvent combinations and additive doping ratios.

In order to verify the universal feasibility of the new fabrication conditions, other Ruddlesden–Popper spacers (PA, BA, PMA, and PEA) were also employed. As shown in the X-ray diffractograms in Fig. S2a (ESI†), the fabricated quasi-2D perovskite films with 〈*n*〉 = 5 all showed the α-phase without the δ-phase. The appearance of a small fraction of PbI_2_ in (PEA)(FA)_4_Pb_5_I_16_ may have been caused by annealing-induced thermal degradation. The bandgaps of the quasi-2D perovskites were in the range of 1.53–1.54 eV (see the Tauc plot in Fig. S2b, ESI†), smaller than those of the MA series (1.60–1.63 eV), which illustrates the advantage over the MA series in absorbing a larger part of the solar spectrum. Therefore, it can be concluded that the synergistic strategies of solvent engineering, additive doping and processing methods successfully fabricated the stable α-phase of FA-based quasi-2D perovskites with multiple L-site spacers.

Fig. 2b shows the UV-vis absorption and steady-state photoluminescence (PL) spectra. The latter were recorded using both front and back excitation with a 405 nm laser source. Considering that the local 2D low-*n* domains (between 530 and 730 nm) existed at both the top and the bottom sides of the film, it implies that the phase distribution in quasi-2D perovskites (PDMA)(FA)_4_Pb_5_I_16_ was not fully regulated. Different model systems were built to describe the phase distribution in the quasi-2D perovskites. From a molecular-scale (local) perspective, 2D domains with different *n* values are interspersed in the 3D domain matrix with a concentration variation in different areas of the film. This may also explain why the PL bands of the 3D domains were observed by both front side and back side excitation. In contrast, in a continuum model, the 3D domains reside mainly on one side, and the 2D low-*n* domains lie mostly on the other side, thus forming a phase gradient in the film thickness direction. Both model systems with regulated phase distributions can induce a sequential energy transfer cascade and boost the photo-induced charge carrier separation and extraction.^46^ But irregular phase distributions, as in the case of the (PDMA)(FA)_4_Pb_5_I_16_ film in Fig. 2b, implies that microstructural domains with different quantum-well structures are randomly distributed in the film, which may lead to blocked and impeded charge transport paths and accelerated charge carrier recombination. Therefore, the phase distribution of FA-based quasi-2D perovskites needs further regulation through manipulating the precursor solution and processing methods.

### 2.2. Manipulation strategies for phase distribution regulation

#### 2.2.1. Additive doping with excess FACl

First, we attempted tuning of the additive to manipulate the phase distribution of the quasi-2D perovskite (PDMA)(FA)_4_Pb_5_I_16_. Although MA in the MACl additive has a low sublimation temperature and is mostly removed after annealing, it can also remain in the film and become involved in A-site cation mixing, shifting the optical bandgap. Therefore, the additive was changed from MACl to FACl, in order to eliminate the impact of the residual MA in the film and maintain the phase purity.

**Fig. 3:**
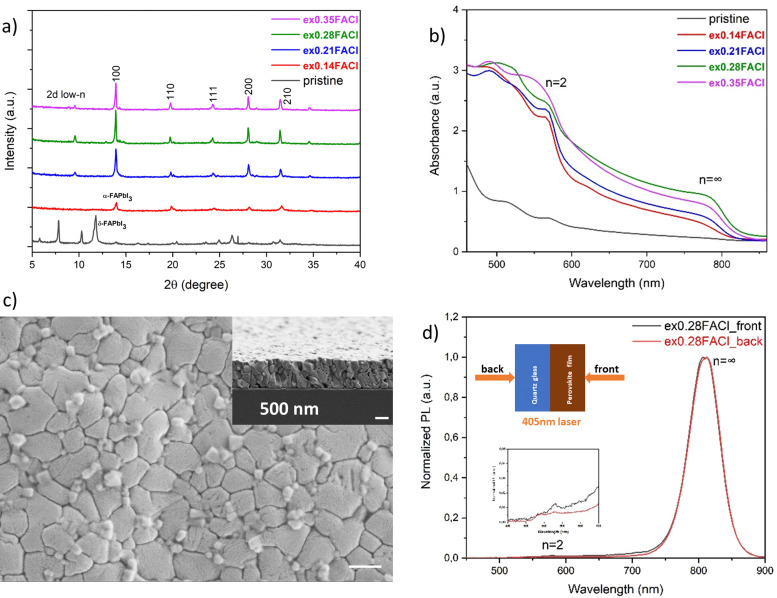
The crystallography, optical properties, surface morphology and phase distribution of quasi-2D perovskites (PDMA)(FA)_4_Pb_5_I_16_ with 14–35 mol% of excess FACl doping. (a) Powder X-ray diffractograms. (b) UV-vis absorption spectra. (c) Surface morphology of the film with 28 mol% of excess FACl addition. The inset is the cross-sectional view, and both scale bars are 500 nm. (d) Steady-state PL of the film with 28 mol% of excess FACl addition, recorded from front (perovskite) and back (substrate) side illuminations at 405 nm. The inset shows the zoomed-in PL signal in the wavelength range between 450 nm and 700 nm.

In principle, doping can be performed in two ways. The first one is to add excess FACl to the precursor solution of the stoichiometric composition of (PDMA)(FA)_4_Pb_5_I_16_. With increasing excess FACl, and the film showed a stable α-phase and increased crystallinity (Fig. 3a). The degree of light absorption was also enhanced, especially for the film with 28 mol% of excess FACl, which had a sharp absorption onset edge representing the 3D domain, and an excitonic band around 580 nm representing the 2D *n* = 2 domain (Fig. 3b). From the steady-state PL data in Fig. 3d, the 3D domain was detected by both front and back excitation, but there was also a weak *n* = 2 PL band from the perovskite side, consistent with the UV-vis absorption spectra. This likely implies that the 2D *n* = 2 domain grew preferentially on the top surface of the film, while the bulk was the 3D domain, hence forming a regulated phase distribution. This result was confirmed by the surface morphology shown in Fig. 3c, where compared to the pristine quasi-2D film (Fig. S3a, ESI†), the grains were larger (average grain size: 500 nm) with some crystals on the surface, especially at grain boundaries. A possible reason may be that with the increasing FACl additive, the crystallization kinetics are retarded, and the large spacer cations at the bottom of the solution have more time to diffuse towards the top surface upon post-annealing. The crystals of 2D *n* = 2 local domains eventually accumulate at the grain boundaries of the 3D domain, passivating defects and suppressing the non-radiative recombination centers. The proposed mechanism of film growth and regulated phase distribution is schematically illustrated at the end of this section.

#### 2.2.2. Substitutional doping with FACl

**Fig. 4:**
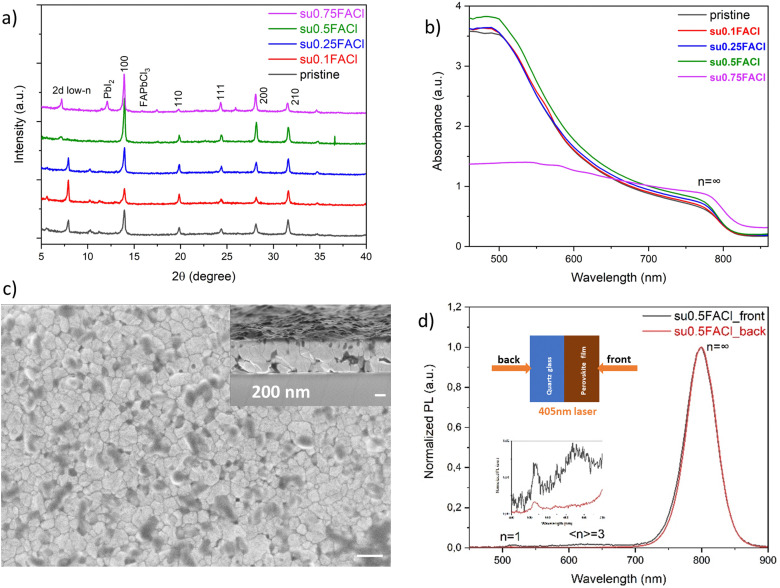
The crystallography, optical properties, surface morphology and phase distribution of quasi-2D perovskites (PDMA)(FA)_4_Pb_5_I_16_ with compensated FACl additive doping. (a) Powder X-ray diffractograms. (b) UV-vis absorption spectra. (c) The surface morphology of the film with 50 mol% of FACl substitution. The inset is the cross-sectional view, and both scale bars are 200 nm. (d) Steady-state PL spectra of the film with 50 mol% of FACl substitution for front (perovskite) and back (substrate) side illuminations at 405 nm. The inset shows the zoomed-in PL signal in the wavelength range between 450 nm and 700 nm.

The other method of doping with the additive is to substitute FAI partially by FACl and to keep the concentration of the FAI + FACl constant, which is called compensated or substitutional doping. Upon replacing 50 mol% of FAI with FACl, both crystallinity and light absorption were slightly enhanced (Fig. 4a and b). An over-dose of 75 mol% of FACl substitution caused the formation of a PbI_2_ phase (2*θ* = 12.8°) and reduced light absorption due to degradation-induced quenching. The crystalline grains were not as large as in the film with 28 mol% of excess FACl, but the vertical growth alignment was more obvious, as evident from the compact and large grains in the cross-sectional view in Fig. 4c, which may facilitate charge transport in the vertical direction. In the steady-state PL in Fig. 4d, there was only one dominating band for the back excitation representing the 3D domain, while for the front excitation, except for the 3D domain, there were also two small emission bands representing 2D *n* = 1 and *n* = 3 domains. In comparison to the pristine film in Fig. 2b, the film with 50 mol% of the substituted FACl additive had a different phase distribution. More specifically, the location of 2D low-*n* domains changed from the bottom side to the top side, embedded in the 3D domain matrix, thus forming a reverse-gradient phase distribution. An illustrated schematic showing film growth and the regulated phase distribution is shown at the end of this section. Recently, the concept of using a 2D spacer as a passivation layer on top of 3D perovskites to increase the stability of 3D perovskites has become popular,^47–52^ but additional post-treatment is usually needed, for instance an extra spin-coating step with 2D spacer ink and an extra annealing step, which might also induce thermal degradation and a non-stoichiometric composition.^53,54^ In contrast, substitutional FACl doping can produce a similar assembled 2D passivation layer without additional post-treatment, making it a facile strategy to protect the film from moisture penetration due to the hydrophobic nature of the large organic spacers that are present. The passivation effect is beyond the scope of this research. Nevertheless, we suggest that a reversed-gradient phase distribution is more suitable for an n–i–p solar cell configuration than for a p–i–n configuration, since the 2D low-*n* domains with higher exciton binding energies can also serve as electron blockers. Therefore, an electron transport layer (ETL) at the bottom interface can extract electrons more efficiently, and a hole transport layer (HTL) at the top interface can do the same for extracting holes. The majority of all quasi-2D perovskites have a normal-gradient phase distribution. Therefore, changing the phase distribution provides an extra handle to improve the photovoltaic performance when considering a whole solar cell architecture.

#### 2.2.3. Spacer mixing of PDMA and ALA

**Fig. 5:**
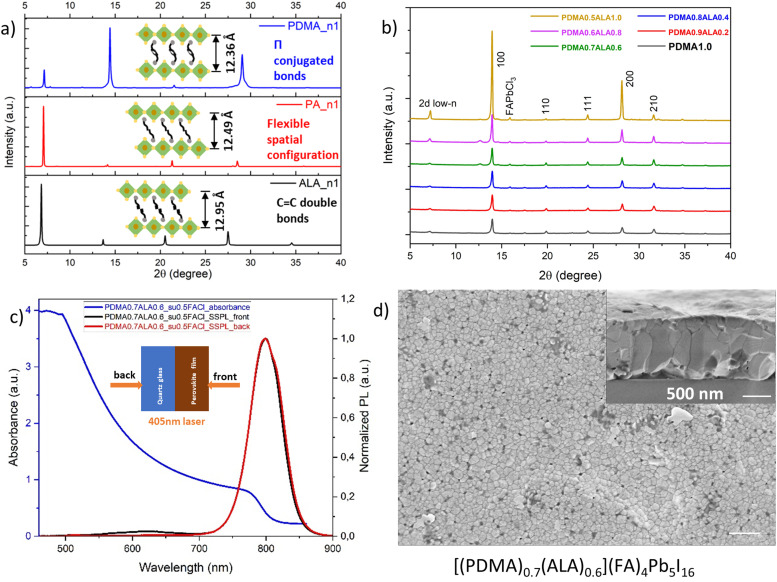
The crystallography, optical properties, phase distribution and surface morphology of quasi-2D perovskites with mixed PDMA and ALA spacers. (a) Powder X-ray diffractograms of pure 2D perovskites (L)_*m*_PbI_4_, where L = PDMA, PA, ALA, and the corresponding interlayer distances. (b) Powder X-ray diffractograms of quasi-2D perovskites [(PDMA)_1−*x*_((ALA)_2_)_*x*_](FA)_4_Pb_5_I_16_ (*x* = 0, 0.1, 0.2, 0.3, 0.4, 0.5) with spacer mixing. (c) UV-vis absorption spectra and steady-state PL from front (perovskite) and back (substrate) sides of the film [(PDMA)_0.7_(ALA)_0.6_](FA)_4_Pb_5_I_16_ with 50 mol% FACl substitution. (c) The surface morphology of the film [(PDMA)_0.7_(ALA)_0.6_](FA)_4_Pb_5_I_16_ with 50 mol% of FACl substitution. Inset is a cross-sectional view, and both scale bars are 500 nm.

Following the principles of spacer mixing in our previous work where we found that different types of spacers should have similar interlayer distances between the [PbI_6_]^4−^ layers and should have an appropriate steric hindrance effect,^45^ spacer mixing was also conducted in this work. First, a new Ruddlesden–Popper spacer ALA was used as the co-spacer mixed together with the Dion–Jacobson spacer PDMA. From the XRD data of pure (ALA)_2_PbI_4_, we calculated its interlayer distance, which is 12.95 Å, slightly larger than that of (PDMA)PbI_4_, which is 12.36 Å (Fig. 5a). The two spacers can be mixed together, and still maintain the perovskite octahedral frameworks and quantum-well structures.^45^ Moreover, the benzyl rings in the PDMA spacer comprises π-conjugated bonds, and the ALA spacer contains C=C double bonds, both of which can form (conjugated) π-bonds, potentially facilitating electron tunnelling through the interlayer,^44^ mitigating the quantum and dielectric confinement, and having a high potential to increase the charge conductivity.^55–59^

Adopting the substitutional FACl doping strategy and substituting 50 mol% of FAI for FACl, a perovskite-phase with the nominal chemical formula [(PDMA)_1−*x*_((ALA)_2_)_*x*_](FA)_4_Pb_5_I_16_ was formed. With an increasing ratio of ALA with respect to PDMA, the quasi-2D perovskites with 〈*n*〉 = 5 all showed the stable α-phase and increasing crystallinity (Fig. 5b). No XRD peak splitting was observed, possibly because ALA is a rigid molecule with C=C double bonds as compared to other Ruddlesden–Popper spacers with C–C single bonds such as PA, which have more degrees of freedom for spatial rearrangement and molecular relaxation. For example, the film [(PDMA)_0.7_(ALA)_0.6_](FA)_4_Pb_5_I_16_ (*x* = 0.3) had a strong light absorption with a sharp absorption onset edge (Fig. 5c). The steady-state PL spectra in Fig. 5c showed a similar reversed-gradient phase distribution as the film (PDMA)(FA)_4_Pb_5_I_16_ with substitutional FACl doping, *i.e.* the 2D low-*n* domains reside on the top side, and the 3D domains reside at the bottom side of the film. Thus, ALA spacer mixing did not change the phase distribution. It was the additive doping method and the different crystallization mechanism that determined the changed evolution of the phase distribution. It is worth noting that the dominant PL band from the back excitation had a small shoulder, implying that it may comprise two separate emission bands very close to each other. A possible reason might be that although the 3D domain normally does not contain spacers, the octahedra in quasi-2D perovskites are locally tilted induced by the two spacers, generating two distinct PL bands. Another possible reason lies on the trap state involved reabsorption and reemission. Furthermore, ALA spacer mixing did change the surface morphology. In contrast to the pristine film (PDMA)(FA)_4_Pb_5_I_16_, which had a rough surface (Fig. S3a, ESI†), and the film (ALA)_2_(FA)_4_Pb_5_I_16_ with crystallites (Fig. S3c, ESI†), the surface morphology of the film [(PDMA)_0.7_(ALA)_0.6_](FA)_4_Pb_5_I_16_ with spacer mixing contained grains resembling the film (PDMA)(FA)_4_Pb_5_I_16_ with the substitutional FACl additive (Fig. 4c). But the vertically aligned grains had grown even larger, spanning the whole film thickness (Fig. 5d), which may facilitate out-of-plane charge carrier transport. The most likely reason is that the ALA spacer can fix the missing bridging to adjacent octahedra left by the bulky PDMA spacers. The better aligned interlayer connections led to an improved vertically aligned crystal growth over larger distances.^45^

#### 2.2.4. Spacer mixing of PDMA and PA

Since the doping method controls the phase distribution, we explored spacer mixing of PDMA and PA based on the pristine film (PDMA)(FA)_4_Pb_5_I_16_ using a DMF/NMP co-solvent system and 10 mol% of excess MACl, and investigated the effect of PA mixing and A-site cation mixing on crystallization and phase distribution manipulation. 2D perovskites with a PA spacer have an interlayer distance of 12.49 Å, very similar to that of PDMA (Fig. 5a). Following prior experience [41], we chose a stoichiometric ratio of spacers ((PDMA)_0.7_(PA)_0.6_) for L-site spacer mixing. For A-site cation mixing, we first explored FA and MA mixing based on the film composition (PA)_2_[(FA)_1−*x*_(MA)_*x*_]Pb_5_I_16_ where *x* = 0, 0.1, 0.3, 0.5, 0.7, 0.9, or 1, since incorporating small-sized MA can help release lattice strain induced by the larger sized FA, while maintaining thermal and structural stability. In Fig. S4a (ESI†), it can be seen that the (100) peak slightly moved to a higher angle with an increasing ratio of MA, implying that the smaller-sized MA incorporated into the perovskite structure. The bandgap changed from 1.53 eV for FA-based (*x* = 0) to 1.61 eV for full MA-based (*x* = 1) quasi-2D perovskites (see the Tauc plot in Fig. S4b, ESI†). This result is consistent with the steady-state PL spectra in Fig. S4c (ESI†), where the FA-based quasi-2D perovskite showed an emission band around 793 nm, and it was blue-shifted to 759 nm upon MA substitution. Thus, the bandgap can also be tuned by A-site cation mixing.

**Fig. 6:**
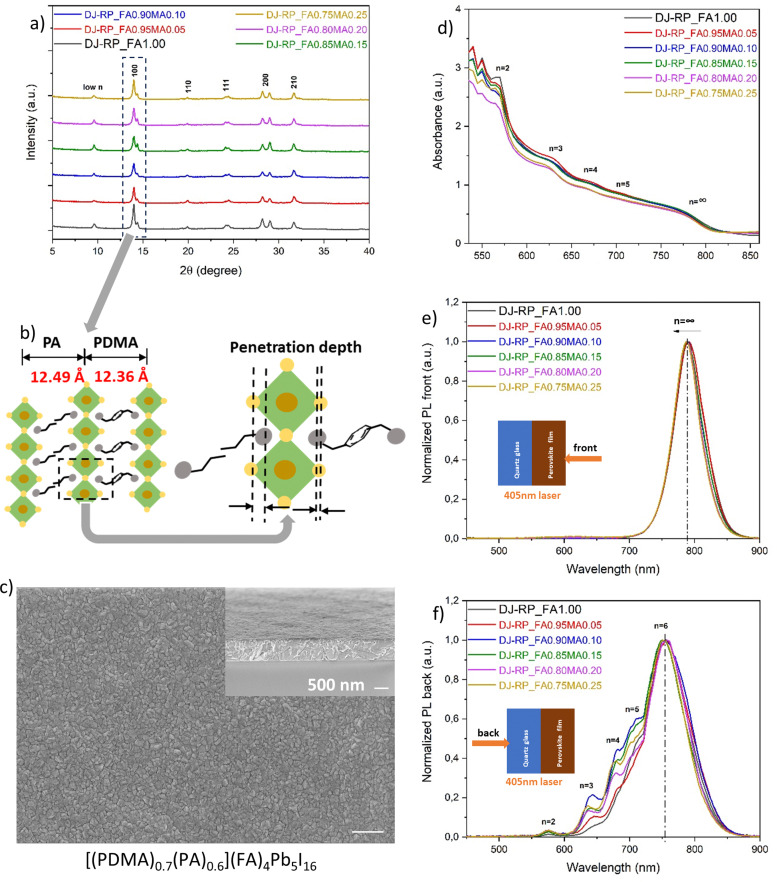
The crystallography, surface morphology, optical properties and phase distribution of quasi-2D perovskites with PDMA and PA spacer mixing and A-site cation mixing. In the labels, “DJ-RP” refers to the mixed system DJ spacer PDMA – RP spacer PA, and “(FA)_1−*x*_(MA)_*x*_” describes the A-site mixing ratio. (a) Powder X-ray diffractograms. (b) Schematic illustration of spacers mixing of PDMA and PA and the corresponding interlayer distance and penetration depth differences. (c) The surface morphology of the film [(PDMA)_0.7_(PA)_0.6_](FA)_4_Pb_5_I_16_. The inset shows the cross-sectional view, and both scale bars are 500 nm. (d) UV-vis absorption spectra. (e) Steady-state PL spectrum, recorded using front (perovskite) side excitation at 405 nm. (f) Steady-state PL spectrum, recorded using back (substrate) side excitation at 405 nm.

L-site spacers and A-site cations were mixed according to the chemical formula [(PDMA)_0.7_(PA)_0.6_][(FA)_1−*x*_(MA)_*x*_]_4_Pb_5_I_16_ (*x* = 0, 0.05, 0.1, 0.15, 0.2, 0.25). Since we want FA to dominate the properties for its smaller bandgap and higher thermal stability, the A-site cation mixing ratio was kept below 25 mol% of MA. Different mixing ratios all showed a stable α-phase, but the XRD data in Fig. 6a also showed a peak split. We assume that, although the two spacers have very similar interlayer distances, the penetration depth of the ammonium heads is slightly different, which probably induces octahedral tilting and splitting of the peak (Fig. 6b). The *d*-spacings from the split peak (6.32 Å and 6.16 Å) match the nuance of the interlayer distances of the two spacers (12.49 Å and 12.36 Å, respectively).

Fig. 6d shows the UV-vis absorption spectra. In addition to the absorption onset, multiple excitonic bands were present, representing multiple microstructural domains of different *n* values in the quasi-2D perovskites. In the steady-state PL spectra from the front excitation, only one dominating emission band in an average of 789 nm was observed, corresponding to the presence of 3D domains (Fig. 6e). The band was slightly blue-shifted from 792 nm to 788 nm upon MA substitution. For the back excitation, the steady-state PL spectra in Fig. 6f shows multiple emission bands of 2D low-*n* domains as expected and consistent with the UV-vis absorption spectra. The results imply that the phase distribution was regulated to a normal-gradient type, compared to the pristine film (PDMA)(FA)_4_Pb_5_I_16_ and its random phase distribution in Fig. 2b. A novel discovery was that the maximum emission at the bottom side of the film occurred around 754 nm. We assume that this belongs to an even higher *n*-valued domain, probably *n* = 6. Layered perovskites with *n* ≥ 6–7 domains have been proposed to be thermodynamically unfavourable or unstable,^60^ but the *n* = 6 domains may have formed because the spacer mixing of PDMA and PA favoured the thermodynamics and lowered the formation energy of higher *n*-valued domains. With 3D domains on the top side and 2D low-*n* domains at the bottom side, this normal-gradient phase distribution with regulated quantum-well structures can facilitate a preferential energy transfer cascade. Taking the film [(PDMA)_0.7_(PA)_0.6_](FA)_4_Pb_5_I_16_ as an example, the surface morphology in Fig. 6c showed fine grains and a relatively smooth surface compared to either the (PDMA)(FA)_4_Pb_5_I_16_ or the (PA)_2_(FA)_4_Pb_5_I_16_ film without spacer mixing (Fig. S3a and b, ESI†), implying that a lower number of defects and improved interfacial contact with another charge transport layer on top were generated by this spacer mixing method.

**Fig. 7:**
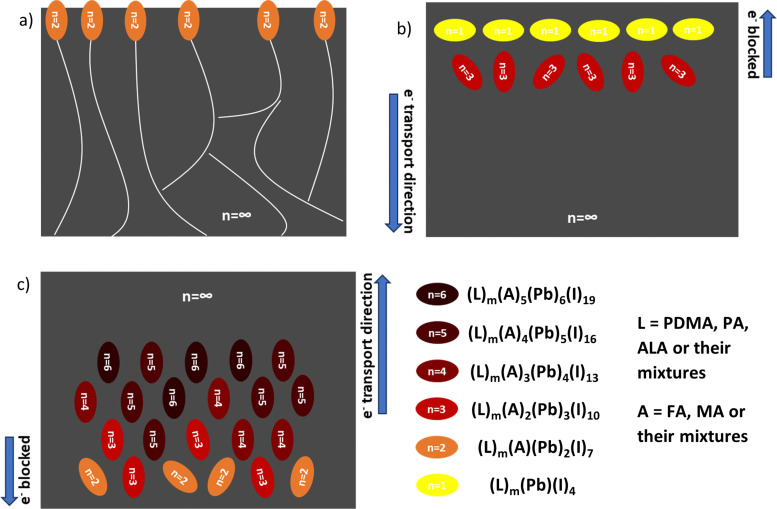
Schematic illustration of the film growth in a cross-sectional view and the regulated phase distributions following different manipulation strategies. (a) 2D low-*n* crystals at the top surface and at the grain boundaries of 3D domains in the (PDMA)(FA)_4_Pb_5_I_16_ film with excess FACl doping. (b) Reversed-gradient phase distribution of the (PDMA)(FA)_4_Pb_5_I_16_ film and [(PDMA)_1−*x*_((ALA)_2_)_*x*_](FA)_4_Pb_5_I_16_ film with FACl substitutional doping. (c) Normal-gradient phase distribution of the [(PDMA)_0.7_((PA)_2_)_0.3_][(FA)_1−*x*_(MA)_*x*_]_4_Pb_5_I_16_ film with 10 mol% excess MACl doping.

Film growth and phase distribution regulation *via* the different manipulation strategies are shown in Fig. 7. With excessive FACl additive doping, the *n* = 2 domain is eventually formed on the top surface of the film, especially at the grain boundaries (Fig. 7a). With substitutional FACl doping, the 2D low-*n* domains (*n* = 1, 3) are formed mainly at the top of the film and embedded in the 3D domain matrix, yielding a revered-gradient (Fig. 7b), whether using only a PDMA spacer or using mixed spacers PDMA and ALA. Finally, when using the mixed spacers PDMA and PA, the 2D domains (*n* = 2–6) are formed mainly at the bottom of the film, yielding a normal gradient (Fig. 7c). All crystallization engineering strategies can regulate the phase distribution of quasi-2D perovskites compared to the pristine film.

## 3. Conclusions

Different manipulation strategies were implemented to regulate the phase distribution of Dion–Jacobson-type quasi-2D perovskites based on the composition of (PDMA)FA_4_Pb_5_I_16_. In general, FACl additive doping changed the crystallization kinetics, leading to 2D low-*n* crystals on the top surface or a reversed-gradient phase distribution, following either an excess doping or a substitutional doping method. Alternatively, mixing with another Ruddlesden–Popper spacer fixed the missing bridging to the adjacent octahedra, regulating the quantum-well structures into a normal gradient phase distribution. Combining both FACl doping and spacer mixing, the film showed properties from both manipulation strategies, *i.e.* a reversed gradient phase distribution and larger vertically aligned grains.

## 4. Experimental section

### 4.1. Materials

Lead (ii) iodide (PbI_2_, 99.999%), methylammonium iodide (MAI, ⩾99%), formamidinium iodide (FAI, ⩾99%), *N*,*N*-dimethylformamide (DMF, 99.8%), dimethyl sulfoxide (DMSO, ⩾99.9%), p-xylylenediamine (PDMA, 99%), allylamine (ALA, 98%), hydroiodic acid 57% (HI, for synthesis), and diethyl ether (⩾99.9%) were purchased from Sigma-Aldrich. Propylamine (PA, ⩾99.0%) was purchased from Fluka. The above-mentioned chemicals were used as received without further purification. Methylammonium chloride (MACl, for synthesis) was purchased from Sigma-Aldrich, and was further dried in a vacuum oven before use.

### 4.2. Synthesis of the PDMAI_2_ powder

First 3.485 g of PDMA was dissolved in ethanol with stirring. Then, 7.37 ml of HI was added and heated at 100 °C in an oil bath, until all precipitates were solidified and all liquids were evaporated. The yellowish clay-like precipitates were purified with diethyl ether, followed by transferring them into a Petri-dish. Finally, the precipitates were dried in a 60 °C vacuum oven for 3 days.

### 4.3. Synthesis of the PAI powder

First, 13.20 ml of HI was added into 8.29 ml of PA, and then heated at 100 °C in an oil bath with continuous stirring, until all precipitates were solidified and all liquids were evaporated. The precipitates were purified with a large amount of diethyl ether until they turned white. This step was followed by transferring them into a Petri-dish. Finally, the precipitates were dried in a 60 °C vacuum oven for 3 days.

### 4.4. Synthesis of the ALAI powder

First, 12.30 ml of cold HI was added dropwise into 7.50 ml of ALA dissolved in 20 ml of IPA in an ice bath and gradually brought back to room temperature. Then, the mixture was heated at 70 °C until the precipitates were solidified and all liquids were evaporated. The precipitates were purified with a large amount of diethyl ether until they turned beige. The precipitates were dried on a 60 °C hotplate for 30 min.

### 4.5. Preparation of precursor solutions

For the 〈*n*〉 = 5 Dion–Jacobson precursor solution, PDMAI_2_, FAI and PbI_2_ powders were dissolved in a stoichiometric molar ratio of 1 : 4 : 5 with a Pb^2+^ concentration of 1M in the mixed solvent DMF : NMP = 9 : 1. Doping with excess FACl was done by increasing the molar FACl:PbI_2_ ratio to a higher value than 4 : 5 while keeping Pb^2+^ in the precursor solution constant. For compensated FACl additive doping, FAI was substituted by FACl in the ratio of FAI : FACl = (1 − *x*) : *x*. For 〈*n*〉 =5, mixed Dion–Jacobson and Ruddlesden–Popper spacers and mixed A-site cation precursor solution, PDMAI_2_, PAI or ALAI, FAI, MAI and PbI_2_ powders were dissolved in the stoichiometric molar ratio (1 − *x*) : 2*x *: 4(1 − *y*) : 4*y *: 5.

### 4.6. Fabrication of quasi-2D perovskite thin films

First, the ultra-flat quartz-coated glass substrate was sequentially cleaned with a detergent, DI water, acetone and IPA in an ultrasonic bath. Then, the substrate was treated by O_2_ plasma. After this, the substrate was transferred to the N_2_ glovebox and attached to the chuck of the spin-coater, and 60 μL of precursor solution was dynamically spin-coated on the substrate with a stepwise spinning program of 1500 rpm for 15 s and 4000 rpm for 20 s. During the second step, 180 μL of chlorobenzene was dropped quickly onto the substrate. The final film was formed by post-annealing at 150 °C on a hotplate for 10 min.

### 4.7. Characterization

Powder X-ray diffraction (XRD) was performed using a Panalytical X’pert Pro Powder diffractometer with Cu Kα_1_ radiation. The UV-vis absorption spectra were recorded using a UV/VIS/NIR PerkinElmer Lambda 950 Spectrometer in a transmittance (*T*) mode, after which the absorbance (*A*) was calculated from *A* = 2 – log *T*. Steady-state photoluminescence was performed using a Blue-Wave Spectrometer from StellarNet Inc with a 405 nm laser from MatchBox series and a FGL435S color filter from Thorlabs. Scanning electron microscopy (SEM) (point resolution) was performed with a Zeiss Merlin HR-SEM equipped with energy dispersive X-ray spectroscopy (EDX) (spatial resolution).

## Author contributions

The manuscript was written through contributions of all authors. All authors gave approval to the final version of the manuscript.

## Data availability

The data supporting this article have been included as part of the ESI.†

## Conflicts of interest

The authors declare no conflicts of interest.

## Supplementary Material

TC-012-D4TC02231A-s001
