# Supplementary material for: Phase distribution regulation of formamidinium-based quasi-2D perovskites through solution engineering
Source: J Mater Chem C Mater. 2024 Aug 22;12(38):15671–81. doi: 10.1039/d4tc02231a (PMC11372761; doi:10.1039/d4tc02231a)
Supplement: TC-012-D4TC02231A-s001 [file TC-012-D4TC02231A-s001.pdf]

Supplementary Information (SI) for Journal of Materials Chemistry C.
This journal is © The Royal Society of Chemistry 2024

Supporting information

Phase Distribution Regulation of Formamidinium-based Quasi-2D Perovskites through Solution Engineering

Xiao Zhang[1], Lisanne Einhaus[2], Annemarie Huijser[2], Johan E. ten Elshof[1]

[1] Inorganic Materials Science Group, MESA+ Research Institute, University of Twente, 7500 AE Enschede, the Netherlands

[2] PhotoCatalytic Synthesis Group, MESA+ Research Institute, University of Twente, 7500 AE Enschede, the Netherlands

Table S1. Orthogonal experiments using different solvent combinations and excess doping fractions for quasi-2D perovskites $(PDMA)(FA)_4Pb_5I_{16}$.

	Without additive	10 mol% MACl	10 mol% NH_4SCN	10 mol% MACl + NH_4SCN	30 mol% MACl
DMF:DMSO = 4:1	δ-phase	Low surface coverage	δ-phase	Low surface coverage	-
DMF:NMP = 9:1	Minor δ-phase	α-phase	Minor δ-phase	α-phase with peak split	α-phase with peak split

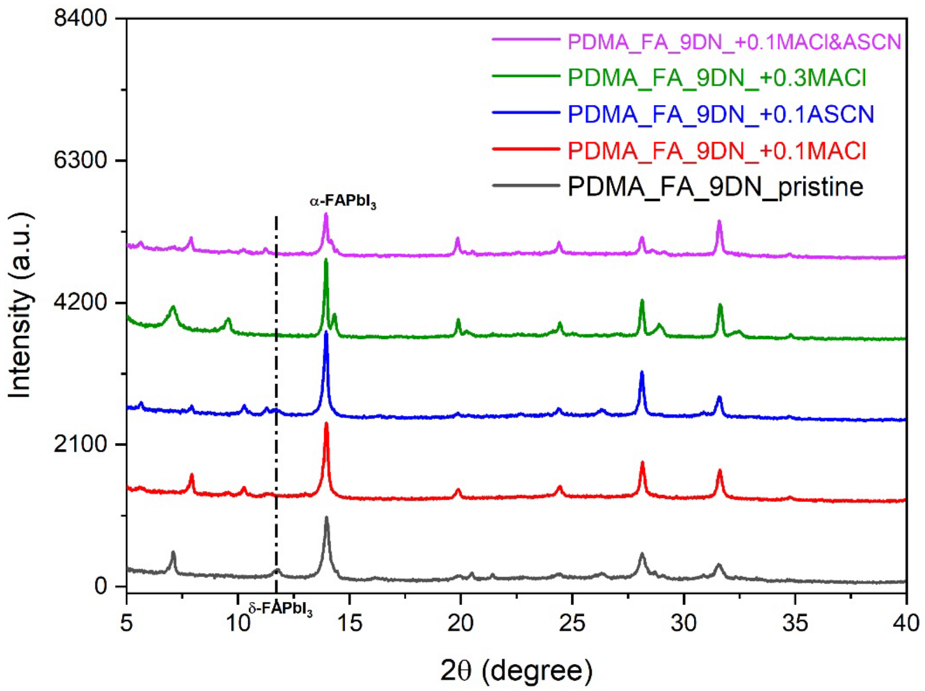

Figure S1. Power X-ray diffractograms of quasi-2D perovskites $(PDMA)(FA)_4Pb_5I_{16}$ with fixed solvent combination of 9:1 (v/v) DMF:NMP and different additive doping ratios.

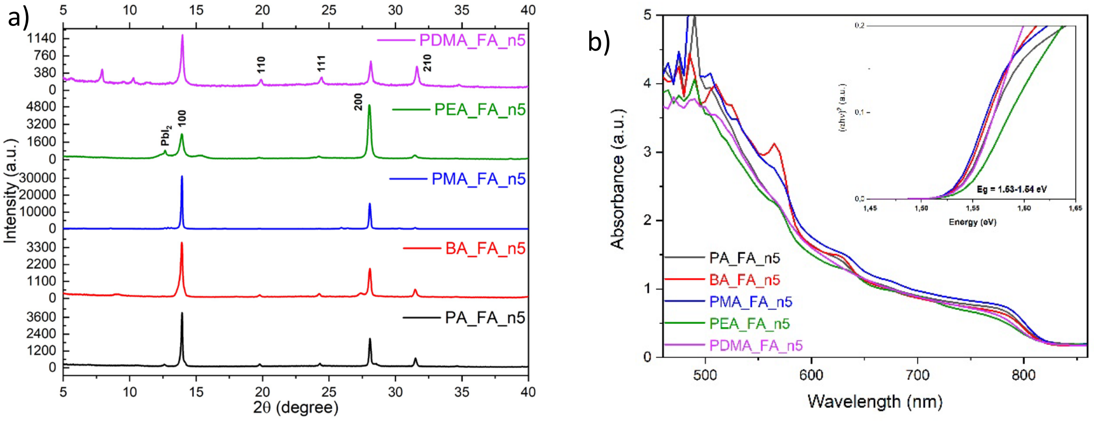

Figure S2. Crystal orientation and optical properties of quasi-2D perovskites $(L)_m(FA)_4Pb_5I_{16}$, L = PA, BA, PMA, PEA, PDMA. (a) Power X-ray diffractograms. (b) UV-Vis absorption spectra (Tauc plot in the inset).

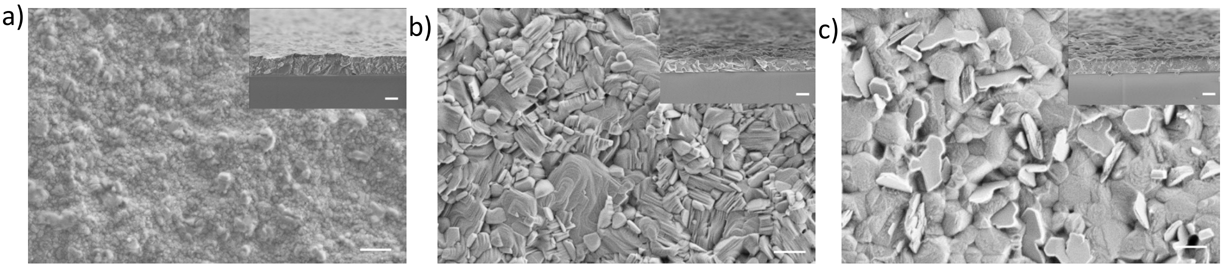

Figure S3. Surface morphologies of the films of $(PDMA)(FA)_4Pb_5I_{16}$, $(PA)_2(FA)_4Pb_5I_{16}$, $(ALA)_2(FA)_4Pb_5I_{16}$, respectively, with solvent ratios of 9:1 (v/v) DMF:NMP, and 10 mol% excess MACl doping. Insets are the cross-sectional views, all scale bares are 500 nm.

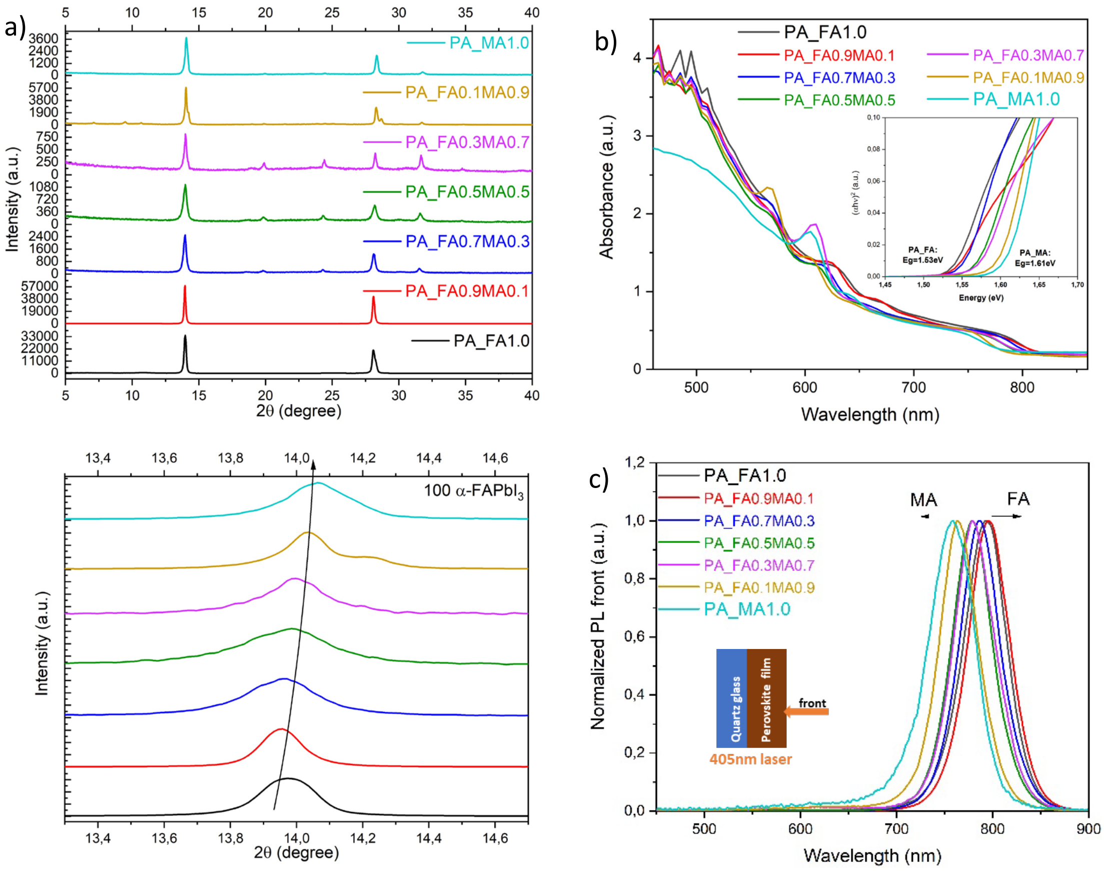

Figure S4. Crystalline orientation, optical properties and phase distribution of quasi-2D perovskites $(PA)_2(FA)_4Pb_5I_{16}$ with A-site cations (FA and MA) mixing. (a) Powder X-ray diffractograms (upper part), and magnified 100 peak (lower part). (b) UV-Vis absorption spectra (Tauc plot in the inset). (c) Steady-state PL from front (perovskite) with a near-UV laser of 405 nm.
